# Bioelectrical Impedance Vector Analysis: A Valuable Tool to Monitor Daily Body Hydration Dynamics at Altitude

**DOI:** 10.3390/ijerph18105455

**Published:** 2021-05-20

**Authors:** Ivo B. Regli, Rachel Turner, Simon Woyke, Simon Rauch, Hermann Brugger, Hannes Gatterer

**Affiliations:** 1 Institute of Mountain Emergency Medicine, Eurac Research, 39100 Bolzano, Italy; Rachel.Turner@eurac.edu (R.T.); Simon.Woyke@i-med.ac.at (S.W.); Simon.Rauch@eurac.edu (S.R.); Hermann.Brugger@eurac.edu (H.B.); Hannes.Gatterer@eurac.edu (H.G.); 2 Department of Anaesthesia and Intensive Care, “F. Tappeiner” Hospital, 39012 Merano, Italy; 3 Department of Anaesthesiology and Intensive Care, Medical University, 6020 Innsbruck, Austria

**Keywords:** bioimpedance, total body water, high altitude, acute mountain sickness, circadian rhythm, intracellular, extracellular

## Abstract

Bioelectrical impedance vector analysis (BIVA) is a method used to estimate variation in body hydration. We assessed the potential of BIVA for monitoring daily body hydration fluctuations in nine healthy, normally active males under matching normoxic (NX) and hypobaric hypoxic (HH) experimental conditions. Furthermore, we aimed to investigate whether changes in BIVA may correspond with the development of acute mountain sickness (AMS). Subjects were exposed in a hypobaric chamber to both NX (corresponding to an altitude of 262 m) and HH conditions corresponding to an altitude of 3500 m during two four-day sojourns within which food, water intake and physical activity were controlled. Bioimpedance and body weight measurements were performed three times a day and medical symptoms were assessed every morning using the Lake Louise score (LLS). Total body water (TBW) was also assessed on the last day of both sojourns using the deuterium dilution technique. We detected circadian changes in vector length, indicating circadian body water variations that did not differ between NX and HH conditions (ANOVA effects: time: *p* = 0.018, eta^2^ = 0.149; interaction: *p* = 0.214, eta^2^ = 0.083; condition: *p* = 0.920, eta^2^ = 0.001). Even though none of the subjects developed AMS, four subjects showed clinical symptoms according to the LLS during the first 24 hours of HH conditions. These subjects showed a pronounced (Cohen’s d: 1.09), yet not statistically significant (*p* = 0.206) decrease in phase angle 6 hours after exposure, which may indicate fluid shift from the intracellular to the extracellular compartment. At the end of each sojourn, vector length correlated with deuterium dilution TBW “gold standard” measurements (linear regression: NX: *p* = 0.002 and r^2^ = 0.756, HH: *p* < 0.001 and r^2^ = 0.84). BIVA can be considered a valuable method for monitoring body hydration changes at altitude. Whether such changes are related to the development of clinical symptoms associated with AMS, as indicated in the present investigation, must be confirmed in future studies.

## 1. Introduction

Bioelectrical impendence analysis (BIA) is a non-invasive tool for assessing body hydration [1,2]. During bioelectrical impedance measurements, an alternating electrical current is delivered and electrical impedance (Z), composed of electrical resistance (R) and reactance (Xc), is quantified. R is determined by the body’s resistive (i.e., opposition to flow of current) elements, and Xc is determined by the body’s capacitive elements; both differ between body fluids and structures [3,4]. Bioelectrical impendence vector analysis (BIVA) analyzes R and Xc data using a vector-based approach, where changes in the vector lengths correspond to total body water variations, and changes in the phase angle (PA) resemble differences in the ratio of extracellular vs. intracellular water [2,5,6,7,8,9,10]. Thus far, BIVA has been used for monitoring body hydration in nephrology [11,12], nutritional sciences [13] in various clinical settings [3,14,15,16], sports science and medicine [7,10,17,18,19,20,21,22,23,24,25,26,27], pediatrics [28] and high-altitude medicine [29,30].

Altitude exposure is known to acutely trigger several physiological processes, which may affect body fluid status [16], e.g., prompt increases in resting ventilation and heart rate [31], as well as direct or indirect adjustment of hormones involved in fluid regulation [32]. Interestingly, circadian oscillations of various vital parameters such as body temperature, breathing rate and blood pressure have been shown to be affected by hypoxia [33]. At sea level, body water variation determined by BIVA follows a circadian rhythm [34,35], but possible variations after acute exposure have previously not been investigated. Thus, the question arises whether prolonged, moderate altitude exposure also notably effects the circadian fluid rhythm. This may be of clinical importance when comparing hydration status under HH conditions to corresponding sea level values throughout the day. Moreover, changes in body hydration have been previously reported to be a risk factor in the development of acute mountain sickness (AMS) [9,36,37,38,39,40,41,42,43,44], even though contrasting data exist [45]. Accordingly, we aimed to assess whether circadian oscillation in TBW dynamics, as determined by BIVA, are influenced by prolonged exposure to HH conditions corresponding to 3500 m. In addition, we intended to investigate whether it would be possible to connect measurable changes in TBW during a 4-day HH sojourn to AMS diagnoses or any singular AMS-related symptoms.

## 2. Materials and Methods

### 2.1. Study Design

The reported experiments were conducted during a project investigating plasma volume (PV) regulation in HH. Detailed description of the overarching experimental approach is provided by Schlittler et al. [32].

Briefly, eleven healthy, young, male lowlanders were recruited as subjects (25 ± 4 years, 181 ± 8 cm, 72 ± 12 kg, body mass index: 22 ± 3 kg/m^2^). Subjects completed two 4-day sojourns in a hypobaric chamber (terraXcube, Bolzano, Italy), one in HH corresponding to an altitude of 3500 m and one in NX (262 m).

The order of the sojourns was randomly assigned to each participant within a crossover design, and a four week ‘wash-out’ period was applied between sojourns. Four days prior and during each sojourn, participants adhered to standardized physical activity, food and fluid intake. Daily fluid intake was 3 L of water plus a cup of coffee, daily caloric intake was 2380 kcal (51% carbohydrate, 13% protein, 36% fat), sodium intake was 100 mmol/day, and potassium intake was 97 mmol/day. Both the timing and content of meals were pre-determined and standardized between conditions. Main meals were served at 07:00, 13:00 and 19:00 each day within the hypobaric chamber. Habitual physical activity was measured with a step counter during the four days preceding the sojourn and step numbers were replicated during the sojourns, if necessary, using a treadmill. During the sojourns, the Lake Louise score (LLS) of each participant was determined every morning [46]. For the data presented in this article, two of the study subjects were excluded due to insufficient compliance with the BIVA protocol during the sojourns.

### 2.2. BIVA Measurements

Bioimpedance measurements were performed using an impedance plethysmograph (BIA 101 BIVA, AKERN SRL, Montacchielo, Italy) with a 250 µA RMS 50 kHz sinusoidal output signal. The device was calibrated using the standard control circuit supplied by the manufacturer with a known impedance (resistance (R) = 383 ohm; reactance (Xc) = 45 ohm). The accuracy of the device was 1% for R and 1% for Xc. Precision of BIA measurements, if performed under standardized conditions, is reported to be very good, with a variability of 1–2% [47,48]. In the present investigation, all measurements have been performed according to the manufacturer’s guidelines, directly prior to breakfast, lunch and dinner. The subjects were in a supine position with their legs and arms by their sides and values were registered after a minimum of 5 min rest. Subjects were required to empty their bladder immediately before assuming the supine position. Prior to the measurement, the skin was cleaned with an alcohol solution and four contact electrodes (BIATRODES, AKERN SRL, Montacchielo, Italy) were placed on the dorsal surface of the right hand and foot according to the manufacturer’s guidelines. Electrode positions were marked with a permanent marker to ensure consistent placement. Electrodes stayed in place during the day, were removed in the evening and, if necessary, were exchanged if the contact was not adequate [9].

Whole body resistance and reactance were assessed for each time point of interest. Data were analyzed applying the previously described BIVA method [2,11,29]. Measurements of resistance (R) and reactance (Xc) were standardized by the height of subjects and expressed in ohm/m (i.e., R/H and Xc/H). The combination of R (i.e., opposition to the flow of an alternating current through intra- and extracellular ionic solution) and Xc (i.e., capacitive component of tissue interfaces, cell membranes and organelles) yields an impedance vector and a corresponding phase angle (geometric relationship = arc tangent of Xc/R expressed in degrees). The length of the vector, calculated as the hypotenuse of individual impedance values, is inversely related to TBW [3,5,17]. The PA is related to the intracellular water pool and the ratio of extracellular to intracellular volumes [6].

### 2.3. Body Weight Measurements

Body weight was measured directly before all bioimpedance measurements and after voiding. Participants were instructed to always wear the same clothing when weighing themselves.

### 2.4. Deuterium-Based Total Body Water Measurements

TBW data measured by deuterium dilution have been previously reported by Schlittler et al. [32]. We used these data, relating to the level of enrichment of deuterium in saliva, to compare BIVA and the deuterium dilution-derived TBW. Briefly, on the evening of the 4th day, before going to bed, the basal saliva sample was collected from each participant. Then, each participant ingested a precisely determined amount of deuterium which was pre-prepared from a stock solution. Ten hours after deuterium ingestion, during which participants abstained from eating or drinking, another saliva sample was collected. Deuterium enrichment of the second saliva sample, relative to the baseline sample, was assessed with isotope ratio mass spectrometry measurements. TBW was derived by calculating the deuterium dilution space while correcting for non-aqueous exchange using the method of Schoeller et al. [49].

### 2.5. Statistical Analysis

Based on the results of a study investigating vector length changes over the course of an 8-day altitude stay (3,830 m) [30], a sample size of *n* = 9 resulted in a power >80% to detect significant vector length changes (two-tailed, assumed r = 0.6). For the calculation, reported changes after 72 h were considered. ANOVA with a repeated measurement design was done using IBM SPSS 26.0 (Armonk, NY, USA). *p* < 0.05 was considered statistically significant. Missing values (*n* = 6, pertaining to different subjects) were replaced with the mean of the respected daytime measurements of the remaining days. Data are shown as mean ± SEM and if there was an adequate effect size (ES, partial eta^2^ or Cohen’s d as indicated in the text). For better legibility, SEM is not shown for body weight data. Linear regression analysis and unpaired t-tests with Welch’s correction comparison were completed with GraphPad Prism 6 (San Diego, CA, USA).

## 3. Results

### 3.1. Circadian Body Fluid Fluctuations at NX and HH

**Figure 1:**
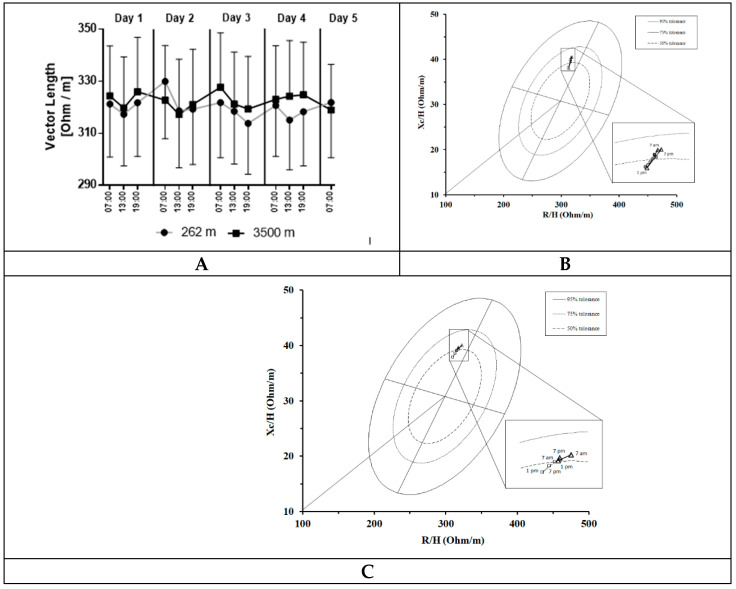
BIVA vector length over time. (**A**) Data represented as mean and standard error of mean (error bars) of nine subjects. ANOVA effects: time: *p* = 0.018, eta^2^ = 0.149; interaction: *p* = 0.214, eta^2^ = 0.083; condition: *p* = 0.920, eta^2^ = 0.001. (**B**) Mean vector shifts during day 1, triangles: altitude, squares: sea level, plotted on the 50%, 75% and 95% tolerance ellipse of the general healthy Italian male population. (**C**) Mean vector shifts during day 4, triangles: altitude, squares: sea level, plotted on the 50%, 75% and 95% tolerance ellipse of the general healthy Italian male population.

During both the NX and HH sojourn, we detected similar circadian rhythms with the longest vectors measured in the morning and shorter ones during the day and in the evening (Figure 1A, ANOVA effects: time: *p* = 0.018, eta^2^ = 0.149; interaction: *p* = 0.214, eta^2^ = 0.083; condition: *p* = 0.920, eta^2^ = 0.001). Except for the first day, the longest vectors were found in the morning and shorter ones at noon and in the evening. During both stays, vector lengths were within the 75% tolerance ellipse of the general healthy Italian male population [50]. Figure 1B,C demonstrate this for days 1 and 4 in the NX and the HH sojourn. Additionally, between conditions (NX and HH) no differences in vector length were identified (ANOVA effect condition: *p* = 0.920, eta^2^ = 0.001).

**Figure 2:**
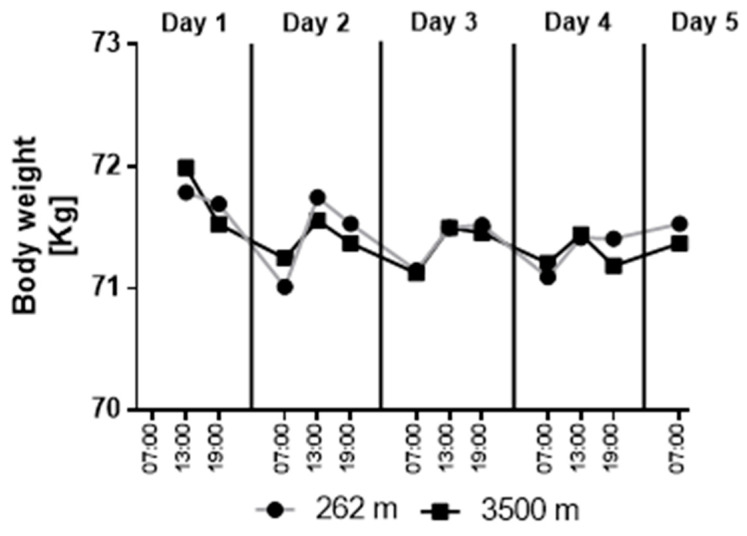
Body weight over time. Data represented as the mean of nine subjects. ANOVA effects: time: <0.001, eta^2^ = 0.265; interaction: *p* = 0.403, eta^2^ = 0.061; condition: *p* = 0.999, eta^2^ = <0.001.

Each participant’s body weight showed a similar dynamic and no differences between conditions (Figure 2, ANOVA effects: time: <0.001, eta^2^ = 0.265; interaction: *p* = 0.403, eta^2^ = 0.061; condition: *p* = 0.999, eta^2^ = <0.001). Similar body weight changes were found in both sojourns, with the lowest weights recorded in the morning and higher values at noon and in the evening. Body weight measurements of the first morning were excluded due to missing data (due to the unfamiliar situation, some subjects forgot to fill in the pre-prepared log on the first morning).

### 3.2. AMS-Related Clinical Symptoms and BIVA Parameters

None of the subjects developed AMS. After 24 hours at 3500 m, four out of nine subjects reported single symptoms according to the LLS (0 < LLS < 3). One participant experienced mild fatigue and weakness and three participants complained about mild headache. During the normobaric sojourn, none of the participants showed any clinical symptoms.

**Figure 3:**
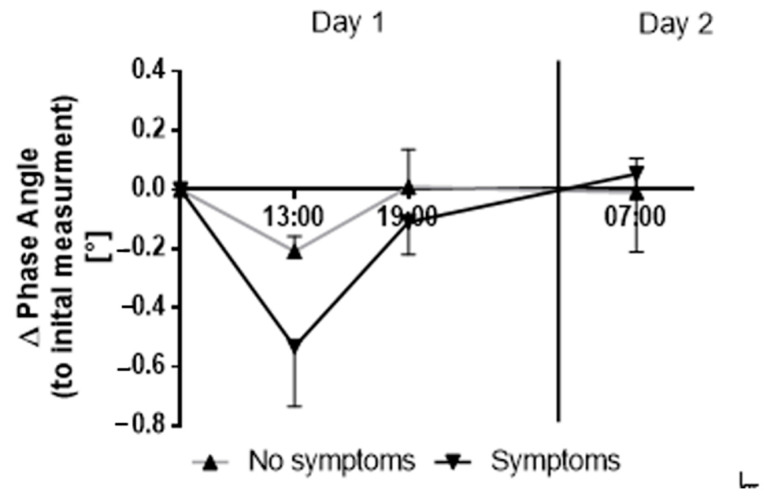
Change in phase angle (PA) from the initial value after simulated ascent. Data from 5 subjects without symptoms are represented with a gray line and upwards-pointing triangles (▲). Data from 4 subjects with symptoms are represented with a black line and downwards-pointing triangles (▼). Data represented as mean and standard error of mean (error bars). Unpaired t-test with Welch’s correction used to analyze changes within the first 6 hours: *p* = 0.206; ES Cohen’s: *d* = 1.09.

The presence of symptoms according to the LLS was accompanied by a more pronounced transient decrease in PA during the first hours (Figure 3). Even though statistical significance was not reached, the increase was most marked 6 hours after simulated ascent (*p* = 0.206, ES (Cohen’s d) = 1.09) but was no longer apparent after 24 hours.

### 3.3. Vector Length and Deuterium Dilution Total Body Water

**Figure 4:**
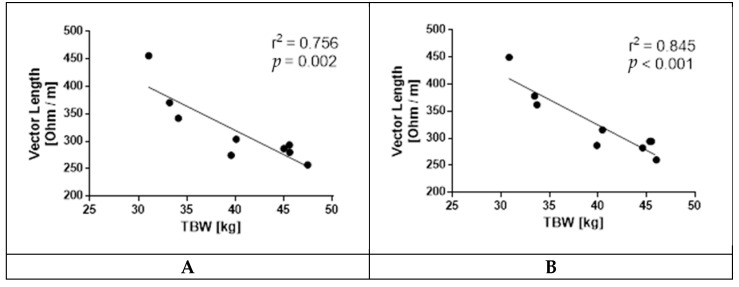
Correlation between total body water (TBW) and vector length. (**A**) Individual data points for deuterium dilution TBW measurements and BIVA measurements taken at the same time at NX. Linear regression: *p* = 0.002 and r^2^ = 0.756. (**B**) Individual data points for deuterium dilution TBW measurements and BIVA measurements taken at the same time at HH. Linear regression: *p* < 0.001 and r^2^ = 0.845.

Similar to vector length, TBW did not differ between conditions (*p* = 0.171 [32]). To test whether BIVA–derived vector lengths are suitable as surrogate markers of TBW status, we correlated the deuterium-based TBW measurements of each participant with their concurrent BIVA measurements. We found a high degree of correlation between these two values in both the NX setting (r^2^ = 0.756 *p* = 0.002) and the HH setting (r^2^ = 0.845, *p* < 0.001) (Figure 4).

## 4. Discussion

Our main finding is that when physical activity, diet and fluid intake are controlled for, TBW circadian rhythm is not influenced by a prolonged hypobaric hypoxic exposure corresponding to 3500 m. Previous studies have revealed that, at sea level, total body water content is subject to circadian fluctuations in healthy volunteers [34,35]. Others report a continuous increase in bioimpedance upon supine positioning of volunteers for 12 hours [51,52]. However, the exact dynamics of this circadian rhythm have not been previously investigated under such controlled conditions, and therefore remain a source of debate.

For most days, our data repeatedly show the longest vector (indicating less fluid) in the early morning and shorter ones (indicating fluid gain) later at ~13:00 in the afternoon and 19:00 in the evening, findings that are similar to those of Buemi et al. [34]. However, no exact pattern can be discerned regarding whether vector length is different at noon or in the evening, probably due to our study subjects undergoing normal physical activity throughout the day instead of being kept in a supine position for prolonged periods of time [34].

Hypoxia influences a variety of hormones involved in fluid regulation [32] and thus it seemed reasonable to hypothesize that TBW circadian rhythm may also be altered. In contrast to our hypothesis, data showed that the circadian rhythm of fluid balance was not affected by exposure to a simulated altitude of 3500 m. Since no fluid volume changes occurred during a prolonged sojourn at this altitude equivalent [32], we may conclude that the level of hypoxia might have been too low to exert significant alterations in TBW and consequently have had little or no effect on fluid circadian rhythm. Whereas, at higher altitudes, body fluid disturbance may be more probable due to potentially higher hormonally induced diuresis and natriuresis, decreased voluntary salt and water intake and increased insensible water loss (respiratory and surface water loss) [53]. Accordingly, Piccoli et al. showed that a significant BIVA vector lengthening compared to sea level can be detected at 5050m [29]. Furthermore, they showed that changes in BIVA vector lengths correlated significantly with changes in body weight, which is in line with our findings. However, it must be considered that the physical activity as well as the degree and duration of hypoxia differed considerably from our study. Furthermore, they showed that plasma osmolality, and serum Na+, K+, Cl- and glucose significantly correlated with BIVA vector length. Moreover, Strapazzon et al. showed that there are significant changes in BIVA vector length upon effortless exposure to an altitude of 3830 m for up to 72 hours [30]. However, here there was no correlation between plasma and serum parameters and BIVA vector length. Although this study was more similar to our study with regard to physical activity as well as the degree and duration of hypoxia, it must still be considered that in the infield setting it is much more difficult to control all the potential confounding parameters. Gatterer et al. showed that there is significant shortening of BIVA vector lengths upon exposure of participants to a FiO_2_ of 0.126 (corresponding to hypoxia at 4500 m) for 12 h [9]. Moreover, there was a significant decrease in serum Na+ and plasma osmolality. Even though, here, the data stem from a controlled environment, it must be considered that the setting of this study is considerably different to ours regarding the degree and duration of hypoxia, the absence of hypobaria and the lack of physical activity. Finally, in women exposed to an altitude of 5050 m for 21 days after a 5 day hike from 2866 m, an absence of changes in vector length has been shown [54]. Here also, differences in the degree of hypoxia, the duration of exposure to hypoxia and the amount of physical activity must be considered. Most importantly, our study is the first to include an NX control sojourn, which allows us to better exclude any confounding factors. Nonetheless, despite our current findings, it could be speculated that above a 3500 m equivalent, fluid circadian rhythm may also be affected by HH, which should be further addressed in future studies. Interestingly, fluctuations in body weight over the course of the HH sojourn were in agreement with changes in vector length. Thus, our data indicate that under strictly controlled and standardized conditions, body weight changes may reflect subtle TBW changes throughout the course of the day, similarly to BIVA. This warrants further consideration in future investigations involving the assessment of TBW, where normally a lack of control dictates that only initial morning body weight measures are considered adequate for fluid status monitoring [55].

In contrast to our expectations, none of the participants developed AMS during the altitude stay [56]. Therefore, it was not possible to link changes in BIVA values to the development of AMS. Nonetheless, participants identified as having singular symptoms according to the LLS after the first 24 hours of HH exposure showed a large, yet not significant, decrease in PA in the hours prior (6 hours; ES = 1.09), which could indicate an initial fluid shift to the extracellular compartment [5,6]. Indeed, body fluid alterations upon ascent to altitude have been previously related to the development of high-altitude illnesses [9,36,37,38,39,40,41,42,57]. Notably, early fluid retention as a result of fluid shifts between intracellular and extracellular water compartments seem to be especially associated with higher AMS occurrence [9,42]. However, in our study, no additional change in vector length was detected in participants showing these symptoms compared to those who remained asymptomatic throughout the exposure. Equally, the initial decrease in PA within the first 6 h was not apparent after 12 h of HH exposure and overall TBW remained unchanged after 4 days of continuous HH exposure. Interestingly, this observation is consistent with previous reports showing that AMS is not associated with changes in TBW [45].

We showed that vector length correlated well with deuterium dilution TBW measurements, widely considered the “gold standard”, confirming that vector length provides specific information on total body water content [3]. The lack of differences in vector length dynamics between the NX and HH sojourn corresponds to the data of the deuterium dilution-based TBW measurements of Schlittler et al. [32].

## 5. Limitations

Some limitations have to be acknowledged. As mentioned, contrary to our expectations, none of the participant suffered from AMS and therefore it was not possible to establish whether early body fluid changes correspond with AMS development in our setting. Nonetheless, we were able to note differences between those participants who developed single symptoms and participants who remained completely asymptomatic. These changes did not reach statistical significance even though effect size was large (ES = 1.09). In this regard, the small sample size and thus the limited statistical power must be recognized as another limitation; therefore confirmation of the present results by further investigation is necessary to draw firm conclusions on this issue.

## 6. Conclusions

In comparison with an NX control, prolonged exposure to a 3500 m equivalent does not alter overall fluid circadian rhythm. In the future, BIVA may present as a practical tool to monitor changes in TBW status under hypobaric hypoxic conditions, if adequate methodological controls are employed relating to diet and fluid intake. Whether changes in TBW are clearly related to the development of clinical symptoms associated with AMS, and are adequately characterized by vector length and PA, remains to be confirmed in future studies.

## Data Availability

The datasets generated and/or analyzed during the current study are available from the corresponding author on reasonable request.
